# Etablierte Klassifikationssysteme der posterioren Malleolus-Fraktur: Eine systematische Literaturübersicht

**DOI:** 10.1007/s00113-022-01162-3

**Published:** 2022-04-08

**Authors:** Elena Müller, Julia Terstegen, Holger Kleinertz, Hanneke Weel, Karl‑Heinz Frosch, Alexej Barg, Carsten Schlickewei

**Affiliations:** 1 Klinik und Poliklinik für Unfallchirurgie und Orthopädie, Universitätsklinikum Hamburg-Eppendorf, Martinistr. 52, 20246 Hamburg, Deutschland; 2 Orthopedics Center, Sint Maartenskliniek, Nijmegen, The Netherlands; 3 Abteilung Unfallchirurgie, Orthopädie und Sporttraumatologie, BG Klinikum Hamburg, Hamburg, Deutschland; 4 Orthopädische Universitätsklinik, Universität von Utah, Salt Lake City, UT USA

**Keywords:** Sprunggelenkfraktur, Systematische Literaturübersicht, Haraguchi, Bartonicek, Mason, Ankle fracture, Systematic literature review, Haraguchi, Bartonicek, Mason

## Abstract

**Hintergrund:**

Frakturen des posterioren Malleolus finden sich bei fast 50 % aller Sprunggelenkfrakturen. Die hohe klinische Relevanz dieser Gelenkfrakturen erklärt sich durch das deutlich schlechtere klinische sowie funktionelle Outcome. Bis heute mangelt es an einer einheitlichen Lehrmeinung bezüglich Klassifikation und Behandlung dieser Frakturen.

**Ziel der Arbeit:**

Intention dieser Arbeit war eine systematische Literaturübersicht über klinische Studien, welche posteriore Malleolus-Frakturen untersucht und mit einer der 3 etablierten Klassifikationen nach Haraguchi, Bartonicek/Rammelt oder Mason klassifiziert haben.

**Material und Methoden:**

Die Datenbank *PubMed* wurde vollständig am 01.07.2021 durchsucht. Nur Publikationen in englischer und deutscher Sprache wurden eingeschlossen. Die systematische Literatursuche wurde entsprechend den aktuellen Kriterien von „Preferred Reporting Items for Systematic Review and Meta-Analyses“ (PRISMA) durchgeführt. Die methodologische Qualität der eingeschlossenen Studien wurde anhand des modifizierten Coleman-Scores quantifiziert.

**Ergebnisse:**

Insgesamt 27 Studien mit insgesamt 2220 Patienten konnten in die systematische Literaturübersicht eingeschlossen werden. Trimalleolarfrakturen zeigten dabei eine deutlich ungünstigere Prognose als andere OSG-Frakturen. Prognostisch entscheidend für das klinische Outcome war v. a. die Qualität der Reposition.

**Diskussion:**

Keine der 3 untersuchten Klassifikationen konnte sich bisher in der Fachliteratur durchsetzen. Speziell im Hinblick auf einen ableitbaren Therapiealgorithmus oder auf eine Prognose hinsichtlich des Outcome sind die untersuchten Klassifikationen schwach oder nicht zu verwenden. Einzig die Klassifikation nach Bartonicek/Rammelt ist geeignet, sich aufgrund des ableitbaren Therapiealgorithmus in der Literatur sowie im klinischen Alltag durchzusetzen.

**Zusatzmaterial online:**

Die Online-Version dieses Beitrags (10.1007/s00113-022-01162-3) enthält zusätzliche Tabellen.

## Abstract (en)

**Background:**

Posterior malleolar fractures are found in almost 50% of all ankle fractures. The high clinical relevance of these joint fractures is explained by the significantly worse clinical and functional outcome. There is still a lack of unified opinion regarding the classification and treatment of these fractures.

**Objective:**

The aim of this article is to provide a systematic literature review of clinical studies that investigated posterior malleolar fractures and classified them using one of the three established classifications according to Haraguchi, Bartonicek/Rammelt, or Mason.

**Material and methods:**

PubMed was searched without time limits. The systematic literature search was performed according to the current criteria of Preferred Reporting Items for Systematic Review and Meta-Analyses (PRISMA). The methodological quality of the included studies was quantified using the modified Coleman score.

**Results:**

A total of 27 studies with a total of 2220 patients were included in this systematic literature review. Trimalleolar fractures showed a significantly less favorable prognosis than other ankle fractures. The quality of reduction was the most important prognostic factor for the clinical outcome.

**Conclusion:**

None of the three classifications examined has become established in the literature. Most of the classifications are weak or should not be used with respect to a derivable treatment algorithm or a prognosis with respect to the outcome. Only the classification according to Bartonicek/Rammelt is suitable to become established in the literature and in clinical practice due to its derivable treatment algorithm.

## Einführung

Frakturen des oberen Sprunggelenks zählen zu den häufigsten knöchernen Verletzungen des Menschen. Im Bereich lasttragender Gelenke präsentiert das Sprunggelenk, mit einer Gesamtprävalenz von 4–9 %, die Region mit den meisten Knochenbrüchen [19, 39]. Knochenbrüche des hinteren Kantenfragments der distalen Tibia, auch Bruch des posterioren Malleolus, oder im deutschsprachigen Raum Fraktur des Volkmann-Dreiecks genannt, können bei etwa 50 % aller Sprunggelenkfrakturen diagnostiziert werden [5, 12, 34, 37, 41, 47, 48]. Die hohe klinische Relevanz posteriorer Malleolus-Frakturen konnten erstmals McDaniel und Wilson im Jahre 1977 nachweisen [28]. Sie beschrieben ein deutlich schlechteres klinisches sowie funktionelles Outcome für Sprunggelenkfrakturen mit Beteiligung des posterioren Malleolus. Ungeachtet des gesteigerten Bewusstseins für die Bedeutung und unabhängig von verbesserten Implantaten bleibt die vergleichsweise schlechtere Prognose von Sprunggelenkfrakturen mit Beteiligung des posterioren Malleolus bis heute bestehen [34, 41, 45]. Trotz intensiver Forschung mangelt es weiterhin an einer einheitlichen Lehrmeinung bezüglich Klassifikation und Behandlung posteriorer Malleolus-Frakturen.

Der Begriff Volkmann-Dreieck, von Earle 1828 in *Lancet* erstbeschrieben und von Volkmann 1875 in seiner Publikation nachweislich nicht erwähnt, ist fest in der deutschen und englischen Literatur verankert [2]. Der Begriff des posterioren Malleolus wurde von Destot erst im Jahre 1911 geprägt [10]. Die erste Empfehlung einer osteosynthetischen Versorgung posteriorer Malleolus-Frakturen ab einer Fragmentgröße von mehr als einem Drittel der Gelenkfläche im seitlichen Röntgenbild postulierten Nelson et al. 1940 [36]. Diese Therapieempfehlung, beruhend auf einem Studienkollektiv von 8 Patienten nach konservativer Therapie und schlechtem Outcome, hielt sich in teilweise leicht abgeänderter Form (ein Viertel bzw. ein Fünftel der Gelenkfläche) bis heute. Erst in den letzten Jahren kam es zu einem schleichenden Paradigmenwechsel [34].

Entscheidend für die Entwicklung neuer Therapieempfehlungen und Klassifikationen war der zunehmende Einsatz der Computertomographie (CT) in der Diagnostik bi- und trimalleolärer Sprunggelenkfrakturen. Mehrere Forschungsgruppen konnten nachweisen, dass eine exakte Größenabschätzung des posterioren Malleolus-Fragments im seitlichen Röntgenbild durch den oftmals irregulären und schrägen Frakturverlauf nur schwer möglich ist [4, 11, 41, 44]. So gilt die CT mittlerweile als Goldstandard für die Diagnostik und Therapieplanung komplexer Sprunggelenkfrakturen. Ergänzend konnten biomechanische In-vitro-Studien nachweisen, dass nicht die Größe des hinteren Kantenfragments, sondern vielmehr die Beteiligung der Inzisur und die Qualität der Reposition für das Ergebnis der Behandlung prognostisch entscheidend sind [23, 34, 45].

Ausschlaggebend für die Relevanz einer Frakturklassifikation im klinischen Alltag sind, neben einer einfachen und nachvollziehbaren Anwendbarkeit, standardisiert ableitbare Empfehlungen bezüglich der Therapie und der damit einhergehenden Prognose. Der flächendeckende Einsatz der CT hat das vorherrschende Bild über die posterioren Malleolus-Frakturen zunehmend verändert. Die neuen Erkenntnisse über Frakturgeometrie und topografische Anatomie verdrängen alte Grundkonzepte, wie die Ein-Drittel-Regel (Fragmentgröße im seitlichen Röntgenbild) nach Nelson [36]. Unterschiedliche Arbeitsgruppen haben diese neu vorliegenden Daten und Parameter gebündelt und analysiert. Auf dem Boden dieser Erkenntnisse haben sich 3 Klassifikationen der posterioren Malleolus-Fraktur etabliert: nach Haraguchi [14], Bartonicek und Rammelt [4] und Mason [27].

Das Ziel dieser Arbeit ist die systematische Übersicht der vorhandenen klinischen Studien, welche eine der 3 etablierten Klassifikationen verwendet haben. Alle 3 Klassifikationen werden detailliert beschrieben sowie deren Vorteile und Nachteile diskutiert.

## Methodik

### Suchalgorithmus

Die wichtigste Datenbank der medizinischen Fachliteratur (*PubMed*) wurde ohne zeitliche Beschränkungen am 01.07.2021 durchsucht. Die Literaturverzeichnisse der eingeschlossenen Arbeiten wurden ebenfalls berücksichtigt. Nur Publikationen in englischer und deutscher Sprache wurden eingeschlossen. Der folgende Suchalgorithmus wurde verwendet: ((post* AND (mall* OR tibia) AND fracture) OR (Volkmann AND fracture)) AND (barton* OR rammelt or haraguchi or mason). Die systematische Literatursuche wurde entsprechend den aktuellen Kriterien von „Preferred Reporting Items for Systematic Review and Meta-Analyses“ (PRISMA) [29] von 2 Personen unabhängig voneinander durchgeführt.

### Auswahl der Studien

Für die finale Analyse wurden in diesem systematischen Review nur klinische Studien berücksichtigt: Kadaverstudien, bildgebende Studien und Übersichtsarbeiten wurden ausgeschlossen. Klinische Studien wurden nur dann eingeschlossen, wenn sie folgende Kriterien erfüllten: 1) Patientenkohorte mit einer chirurgischen Behandlung einer posterioren Malleolus-Fraktur und 2) klare und eindeutige Klassifizierung von posterioren Malleolus-Fraktur mittels einer oder mehrerer Klassifikationen (Haraguchi, Bartonicek/Rammelt und/oder Mason). Die Auswahl der Studien wurde von 2 Personen unabhängig voneinander durchgeführt.

### Bewertung der methodologischen Qualität von eingeschlossenen Studien

Die methodologische Qualität von eingeschlossenen Studien wurde anhand des modifizierten Coleman-Fragebogens durchgeführt (Version online einsehbar) [9]. Die Studien wurden dabei nach folgenden Kriterien bewertet: Größe der Patientenkohorte, mittlere Nachuntersuchungszeit, Anzahl von durchgeführten chirurgischen Eingriffen, Studientyp, diagnostische Methoden, Beschreibung des chirurgischen Eingriffs sowie der postoperativen Rehabilitation sowie die Methodik der Nachuntersuchung. Alle eingeschlossenen Studien wurden von 2 Personen anhand des modifizierten Coleman-Scores analysiert.

### Statistische Analyse

Im Hinblick auf die ausgewerteten Patientenkohorten wurden gewichtete Mittelwerte berechnet. Die lineare Regressionsanalyse wurde verwendet, um die zeitliche Entwicklung des Coleman Scores zu untersuchen. Alle Berechnungen wurden mit IBM SPSS Statistics Version 26.0 (Fa. IBM, Armonk, NY, USA) durchgeführt.

## Ergebnisse

**Figure 1:**
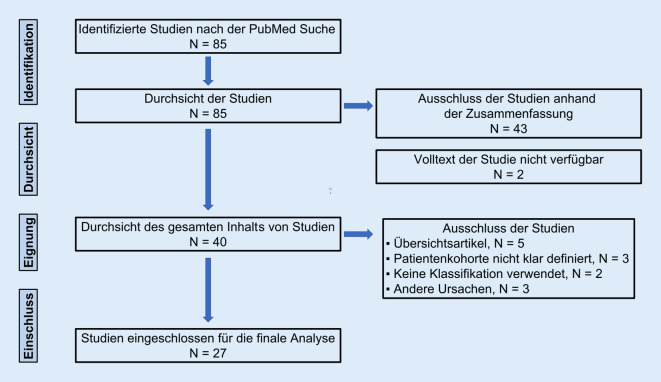

Insgesamt konnten 27 klinische Studien in diese systematische Literaturübersicht eingeschlossen werden (Abb. 1), welche zwischen 2006 und 2021 publiziert wurden (Gesamtübersicht online). Insgesamt wurden 2220 Patienten mit einem gewichteten mittleren Alter von 48,5 Jahren untersucht, hierunter 951 Frauen und 1033 Männer (4 Studien gaben keine Geschlechterverteilung an).

Der Mittelwert des Coleman-Scores lag bei 83,3 ± 16,5 Punkten (35 bis 90 Punkte) (Teil A: 56,0 ± 13,4 Punkte und Teil B: 27,3 ± 5,4 Punkte). Der gewichtete Mittelwert (nach der Anzahl von eingeschlossenen Patienten) des insgesamt ermittelten Coleman-Scores lag bei 75,6 Punkten. Die lineare Regressionsanalyse zeigte, dass der Wert des Coleman-Scores während des Publikationszeitraums zwischen 2006 und 2021 konstant blieb und keine erkennbaren Änderungstendenzen aufwies (R^2^ = 0,014; *p* = 0,946).

### Klassifikation nach Haraguchi

**Figure 2:**
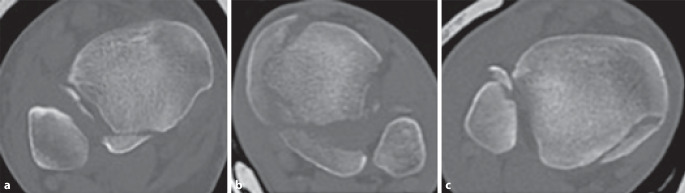

Die erste CT-basierte Klassifikation erstellten Haraguchi et al. im Jahre 2006 an einem Patientenkollektiv von 57 Patienten (medianes Alter 43 Jahre) [14]. Das primäre Ziel ihrer Studie war es, die Pathoanatomie von Frakturen des posterioren Malleolus anhand der präoperativen CT-Bildgebung genauer auszuwerten. Aus den gewonnenen Erkenntnissen leiteten sie 3 Frakturtypen ab (Tab. 1; Abb. 2a–c).

**Table 1:** 

	Beschreibung	Häufigkeit (in %)
Typ I	Posterolaterale Schrägfraktur (dreieckförmiges Fragment der posterolateralen distalen Tibia) (Abb. 2a)	67
Typ II	Quere, nach medial ausstrahlende Fraktur (von der Notch der Fibula bis zum Malleolus medialis verlaufend, meist aus 2 Fragmenten bestehend) (Abb. 2b)	19
Typ III	Kleine (mehr-)fragmentäre schalenförmige Fraktur der dorsalen Tibiakante (Abb. 2c)	14

Primär wurden für alle Patienten die präoperativen Röntgenaufnahmen des OSG im anteroposterioren (a.-p.), bei 20°-Innenrotation („Mortise“-Aufnahme) sowie im lateralen Strahlengang entsprechend dem Unfallmechanismus nach Lauge-Hansen klassifiziert. Haraguchi et al. beschrieben 44 Frakturen vom Supinations-Eversions-Typ, 7 Frakturen vom Pronations-Eversions-Typ sowie 6 Verletzungen vom Pronations-Abduktions-Typ. Die Auswertung der CT (2–3 mm Schichtdicke) erfolgte axial auf Höhe der tibialen Gelenkfläche. Gemessen wurde die anteilige Frakturgröße des posterioren Malleolus im Verhältnis zur gesamten Gelenkfläche. Zudem bestimmten sie den Winkel zwischen der Malleolengabel und der Hauptfrakturlinie.

Die Fragmentgröße der Haraguchi-Typ-I-Frakturen belief sich im Durchschnitt auf 11,7 %, die der Haraguchi-Typ-II-Frakturen auf 29,8 % der tibialen Gelenkfläche. Die durchschnittliche Größe der Haraguchi-Typ-III-Frakturen konnte aufgrund z. T. kleinster Fragmente nicht abschließend bestimmt werden. Der gemessene Winkel zwischen der Malleolengabel und der Hauptfrakturlinie variierte stark zwischen 9° und 40°. Aus der hohen Varianz der Winkel und des stark divergierenden Frakturverlaufs schlussfolgerten Haraguchi et al., dass die CT die präoperative Röntgendiagnostik als Goldstandard zur präoperativen Planung ablösen sollte. Weitere klinische Relevanz sahen sie in der Tatsache, dass Haraguchi-Typ-II-Frakturen, deren Größe eine operative Stabilisierung indizierten, in den medialen Malleolus einstrahlen und somit über einen posteromedialen Zugang adressiert werden sollten. Eine Empfehlung zur operativen Stabilisierung von Haraguchi-Typ-I-Frakturen sprachen sie lediglich bei verbliebener Gelenkstufe nach Fixierung des medialen und lateralen Malleolus aus. Einschränkend muss darauf hingewiesen werden, dass die Ableitung therapeutischer Empfehlungen primär nicht Ziel der durch Haraguchi et al. durchgeführten Studie war. Bereits in der Originalarbeit empfahlen sie bezüglich therapeutischer Empfehlungen weiterführende CT-basierte Studien [14]. Bisher wurde die Klassifikation nach Haraguchi in 17 von 27 eingeschlossenen Studien verwendet (Tab. 2).

**Table 2:** 

Studie	Patienten	Prädiktive Bedeutung
Bali et al., 2017 [1]	*15 Patienten*	Posteromedialer Zugang am OSG eignet sich gut für die Versorgung von Typ II-Haraguchi-Fx
	Typ II: 15 (100 %)	
Blom et al., 2019 [7]	*73 Patienten*	Typ II: schlechteres Outcome (FAOS, Symptome, Schmerz, ADL)
	Typ I: 20 (27 %)	
	Typ II: 21 (29 %)	
	Typ III: 32 (44 %)	
Blom et al., 2020 [6]	*70 Patienten*	2 Jahre FU Typ I: Qualität der Rekonstruktion der tibialen Gelenkfläche als prädiktiver Faktor Typ II: schlechterer FAOS als die Typen I und III Typ III: Qualität der Syndesmosenstabilisierung als prädiktiver Faktor
	Typ I: 23 (33 %)	
	Typ II: 22 (31 %)	
	Typ III: 25 (36 %)	
Haraguchi et al., 2006 [14]	*57 Patienten*	k. A.
	Typ I: 38 (67 %)	
	Typ II: 11 (19 %)	
	Typ III: 8 (14 %)	
He et al., 2020 [15]	*34 Patienten*	k. A.
	Typ I: 20 (59 %)	
	Typ II: 4 (12 %)	
	Typ III: 10 (29 %)	
Hendrickx et al., 2019 [16]	*34 Patienten*	k. A.
	Typ I: 33 (97 %)	
	Typ II: 1 (3 %)	
	Typ III: 0 (0 %)	
Huang et al., 2018 [18]	*42 Patienten*	k. A.
	Typ I: 42 (98 %)	
	Typ II: 0 (0 %)	
	Typ III: 1 (2 %)	
Mangnus et al., 2015 [25]	*45 Patienten*	k. A.
	Typ I: 13 (29 %)	
	Typ II: 15 (33 %)	
	Typ III: 17 (38 %)	
Meijer et al., 2015 [31]	*31 Patienten*	Typ II: Tendenz zur Falscheinschätzung der posteromedialen Beteiligung
	Keine genauen Angaben bezüglich der Subklassifikation	
Meijer et al., 2016 [30]	*31 Patienten*	k. A.
	Keine genauen Angaben bezüglich der Subklassifikation, es wird jedoch erwähnt, dass die Typen I und II gleichmäßig eingeschlossen wurden	
Mertens et al., 2020 [32]	*46 Patienten*	Klinische Outcomes (AOFAS-Score) von Typen I–III besser werdend
	Typ I: 23 (50 %)	
	Typ II: 20 (43 %)	
	Typ III: 3 (7 %)	
Mitchell et al., 2019 [33]	*42 Patienten mit Tibiaschaft-Fx und PMF*	k. A.
	Typ I: 41 (93 %)	
	Typ II: 3 (7 %)	
	Typ III: 0 (0 %)	
Quan et al., 2021 [40]	*95 Patienten*	k. A.
	Typ I: 66 (69 %)	
	Typ II: 19 (20 %)	
	Typ III: 10 (11 %)	
Sun et al., 2021 [43]	*32 Patienten*	Größe des „Die-punch“-Fragments ohne signifikanten Einfluss auf die postoperativen Ergebnisse
	Typ I: 20 (63 %)	
	Typ II: 12 (38 %)	
	Typ III: 0 (0 %)	
Yang et al., 2020 [50]	*27 Patienten*	k. A.
	Typ II: 27 (100 %)	
Yi et al., 2018 [51]	*107 Patienten*	k. A.
	Typ I: 76 (71 %)	
	Typ II: 30 (28 %)	
	Typ III: 1 (1 %)	
Yu et al., 2021 [52]	*76 Patienten*	k. A.
	Typ I: 76 (100 %)	

In keiner der Studien wurden Angaben zu Inter‑/Intra-Observer-Reliabilität gemacht

*ADL* „activities of daily living“, *AOFAS* American Orthopaedic Foot and Ankle Society,* FAOS* Foot and Ankle Outcome Score,* FU* Follow-up,* Fx* Fraktur*, k.* *A.* keine Angaben, *OSG* oberes Sprunggelenk, *OR* Observer-Reliabilität

### Klassifikation nach Bartonicek/Rammelt

**Figure 3:**
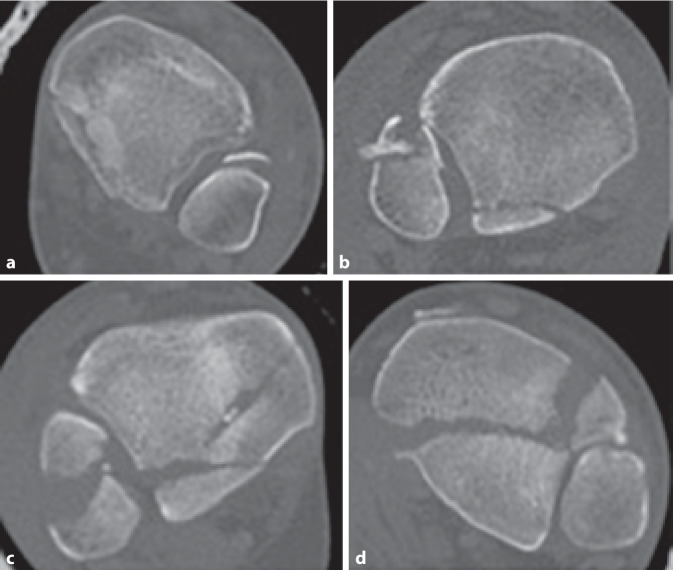

Aufgrund des fortbestehenden Mangels an einer einheitlichen Klassifikation sowie entsprechenden Therapieempfehlungen für Frakturen des posterioren Malleolus führten Bartonicek/Rammelt et al. im Jahre 2015 eine weitere CT-basierte Analyse der Frakturanatomie an einem wesentlich größeren Patientenkollektiv durch [4]. In die Studie konnten 141 Patienten (medianes Alter 49 Jahre) eingeschlossen werden. Nach Auswertung der erhobenen Daten definierten sie für die posteriore Malleolus-Fraktur 5 verschiedene Frakturtypen (Tab. 3; Abb. 3a–d).

**Table 3:** 

	Beschreibung	Häufigkeit (in %)
Typ 1	Fraktur außerhalb der (intakten) Inzisur (Fibula-Notch) (Abb. 3a)	8
Typ 2	Posterolaterales Fragment mit Beteiligung der Inzisur (Fibula-Notch) (Abb. 3b)	52
Typ 3	Zweiteiliges, posteromediales Fragment mit Beteiligung des Innenknöchels (Abb. 3c)	28
Typ 4	Großes posterolaterales dreieckförmiges Fragment (Beteiligung > ein Drittel der Inzisur) (Abb. 3d)	9
Typ 5	Irregulär, osteoporotisch, keinem der anderen 4 Subtypen zuzuordnen	3

Primär erfolgten präoperative Röntgenaufnahmen des OSG (a.-p., Mortise und lateraler Strahlengang) sowie im Anschluss eine erweiterte Bildgebung mittels CT des Sprunggelenks mit axialer, sagittaler und koronarer Rekonstruktion. Die Schichtdicke belief sich auf 1–2 mm. In 64,5 % der Fälle konnten zudem 3D-Rekonstruktionen erstellt werden. Anhand der Röntgenbilder erfolgten eine Auswertung und Klassifikation der Fibulafraktur, einer etwaigen (Sub‑)Luxationsstellung sowie der Art der medialen Verletzung (Fraktur, ligamentäre Verletzung). CT und 3D-CT wurden bezüglich der Größe und Form des posterioren tibialen Kantenfragments ausgewertet. Ein den gesamten Innenknöchel einschließender Frakturverlauf wurde als Pilon-tibiale-Fraktur definiert und aus der Studie ausgeschlossen.

Bartonicek/Rammelt-Typ-2-Frakturen umfassten im Durchschnitt 14 % der tibialen Gelenkfläche sowie ein Viertel bis ein Drittel der Inzisur. In dieser Gruppe konnten v. a. Fibulafrakturen Typ Weber C nachgewiesen werden. Bartonicek/Rammelt-Typ-3-Frakturen entstanden durch kombinierte Kompressions- und Scherkräfte und zeigten eine durchschnittliche Größe von 24 % der tibialen Gelenkfläche. In dieser zweiteiligen Fraktur war das posteromediale Fragment stets größer als das posterolaterale. Bartonicek/Rammelt-Typ-4-Frakturen wurden v. a. durch Kompressionskräfte verursacht und traten mehrheitlich bei Frauen (medianes Alter 61 Jahre) auf. Das mediane Alter der Frakturtypen 1–3 lag mit 46 bis 49 Jahren deutlich darunter. Zudem konnten bei Bartonicek/Rammelt-Typ-4-Frakturen mehrheitlich Weber-B-Frakturen der Fibula nachgewiesen werden.

Die Auswertung der sagittalen CT-Rekonstruktionen zeigte, dass alle Frakturen, inklusive der Frakturen außerhalb der Inzisur, Teile der Gelenkfläche der Tibia betrafen, häufig mit einer Impression der Gelenkfläche im Bereich der Hauptfrakturlinie. Die Zahl der Fälle mit einer tibiotalaren (Sub‑)Luxation, die Größe des Fragments, die vertikale Ausdehnung des Fragments ebenso wie das Ausmaß der Inzisurbeteiligung nahmen mit den Frakturtypen (1–4) zu, was eine Zunahme der Verletzungsschwere impliziert. Abschließend formulierten die Autoren Empfehlungen zur Therapie, inklusive Zugangswahl, anhand der dargestellten Klassifikation (Tab. 4). Diese Empfehlungen fanden bis heute in 11 klinischen Studien Verwendung (Tab. 5).

**Table 4:** 

Frakturtyp	Therapieempfehlung	Zugang
Typ 1	Konservativ	–
Typ 2	Individuelles Vorgehen nach Fragmentgröße und -dislokation, Stufen in der Inzisur, Beteiligung des Malleolus medialis	Posterolateral
Typ 3	Individuelles Vorgehen nach Fragmentgröße und -dislokation, Stufen in der Inzisur, Beteiligung des Malleolus medialis	Posteromedial
Typ 4	Immer operativ	Posterolateral

**Table 5:** 

Studie	Patienten	Prädiktive Bedeutung
Bartonicek et al., 2015 [4]	*141 Patienten*	k. A.
	Typ 1: 11 (8 %)	
	Typ 2: 74 (52 %)	
	Typ 3: 39 (28 %)	
	Typ 4: 13 (9 %)	
	Typ 5: 4 (3 %)	
Bartonicek et al., 2019 [3]	*37 Patienten*	k. A.
	Typ 1: 5 (14 %)	
	Typ 2: 18 (49 %)	
	Typ 3: 11 (30 %)	
	Typ 4: 3 (7 %)	
	Typ 5: 0 (0 %)	
Blom et al., 2020 [6]	*70 Patienten*	Schlechtere postoperative Ergebnisse mit Zunahme der Verletzungsschwere im 2‑Jahres-FU (FAOS)
	Typ 1: 16 (23 %)	
	Typ 2: 30 (43 %)	
	Typ 3: 22 (31 %)	
	Typ 4: 2 (3 %)	
	Typ 5: 0 (0 %)	
Hendrickx et al., 2019 [16]	*34 Patienten*	k. A.
	Typ 1: 0 (0 %)	
	Typ 2: 21 (64 %)	
	Typ 3: 0 (0 %)	
	Typ 4: 12 (36 %)	
	Typ 5: 0 (0 %)	
Kostlivy et al., 2020 [22]	*15 Patienten*	k. A.
	Typ 1: 2 (13 %)	
	Typ 2: 9 (60 %)	
	Typ 3: 4 (27 %)	
	Typ 4: 0 (0 %)	
	Typ 5: 0 (0 %)	
Maluta et al., 2021 [24]	*46 Patienten*	Schlechtere Ergebnisse mit Zunahme der Verletzungsschwere nach 2‑Jahres-FU (AOFAS-Score, OMAS)
	Typ 1: 22 (48 %)	
	Typ 2: 18 (39 %)	
	Typ 3: 6 (13 %)	
	Typ 4: 0 (0 %)	
	Typ 5: 0 (0 %)	
Mertens et al., 2020 [32]	*46 Patienten*	Typ 1 zeigt bessere postoperative Ergebnisse als Typ 2 (AOFAS-Score)
	Typ 1: 2 (4 %)	
	Typ 2: 17 (37 %)	
	Typ 3: 13 (29 %)	
	Typ 4: 14 (30 %)	
	Typ 5: 0 %	
Neumann und Rammelt, 2021 [37]	*100 Patienten*	Kein signifikanter Unterschied bei postoperativen Ergebnissen zwischen Verletzungen der Typen 1–4 (OMAS, FFI, AOFAS-Score)
	Typ 1: 7 (7 %)	
	Typ 2: 35 (35 %)	
	Typ 3: 25 (35 %)	
	Typ 4: 23 (23 %)	
	Typ 5: 0 (0 %)	
Sultan et al., 2020 [42]	*247 Patienten*	Typ-3-Fx haben mit 70 % am häufigsten ein interfragmentäres freies Fragment
	Typ 1: 22 (9 %)	
	Typ 2: 122 (49 %)	
	Typ 3: 54 (22 %)	
	Typ 4: 49 (20 %)	
	Typ 5: 0 %	
Sun et al., 2021 [43]	*32 Patienten*	Größe des „Die-punch“-Fragments ohne signifikanten Einfluss auf die postoperativen Ergebnisse
	Typ 1: 0 (0 %)	
	Typ 2: 0 (0 %)	
	Typ 3: 0 (0 %)	
	Typ 4: 32 (100 %)	
	Typ 5: 0 (0 %)	
Yi et al., 2018 [51]	*107 Patienten*	k. A.
	Typ 1: 1 (1 %)	
	Typ 2: 50 (47 %)	
	Typ 3: 30 (28 %)	
	Typ 4: 26 (24 %)	
	Typ 5: 0 %	

In keiner der Studien wurden Angaben zu Inter‑/Intra-OR gemacht

*AOFAS* American Orthopaedic Foot and Ankle Society, *FAOS* Foot and Ankle Outcome Score, *FFI* Foot Function Index,* FU* Follow-up,* Fx* Fraktur, *k.* *A.* keine Angaben, *OMAS* Olerud-Molander Ankle Score, *OR* Observer-Reliabilität

### Klassifikation nach Mason

**Figure 4:**
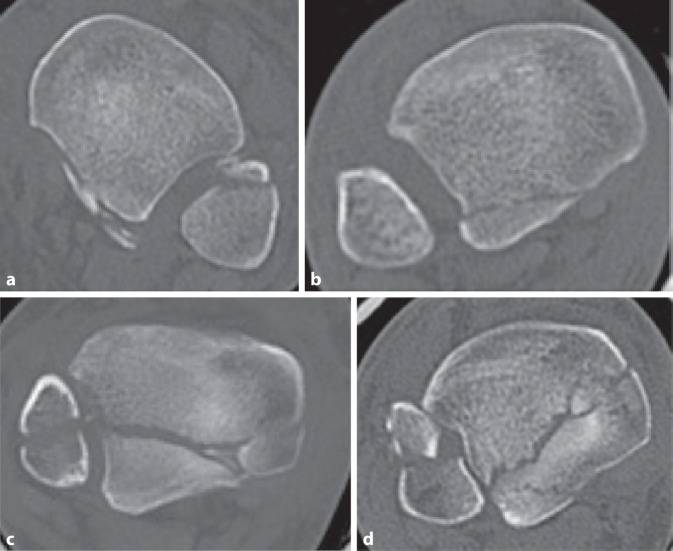

Im Jahre 2017 wollten Mason et al. an einem Studienkollektiv von 121 Patienten (medianes Alter 48 Jahre) die Begleitverletzungen bei Frakturen des posterioren Malleolus in Zusammenschau mit der Klassifikation nach Haraguchi et al. untersuchen [27]. Da diese jedoch den Verletzungsmechanismus nicht abbildet, entwickelten sie eine eigene, in der Schwere der Verletzung aufsteigende Klassifikation (Tab. 6; Abb. 4a–c).

**Table 6:** 

	Beschreibung	Häufigkeit (in %)
Typ 1	Kleines extraartikuläres Fragment (Abb. 4a)	34
Typ 2	Posterolaterales, dreieckförmiges Fragment	45
	2A: posterolaterales, dreieckförmiges Fragment mit Einstrahlung in die Inzisur (Abb. 4b)	55
	2B: zweites posteromediales Fragment (Abb. 4c)	45
Typ 3	Querer Frakturverlauf, die gesamte dorsale tibiale Gelenkfläche umfassend (= posteriore Pilonfraktur) (Abb. 4d)	21

Die Auswertung der CT erfolgte in der axialen Ebene 5 mm proximal der tibialen Gelenkfläche sowie in der sagittalen Ebene 1 cm medial der Inzisur. Die Definition der verschiedenen Frakturtypen basierte auf den hypothetischen Unfallmechanismen. Als Ursache für Mason-Typ-1-Frakturen definierten sie eine Avulsion des posterioren inferioren tibiofibularen Ligaments, bedingt durch auf den plantar-flektierten, entlasteten Fuß wirkende Rotationskräfte. Als Unfallmechanismus der Mason-Typ-2A-Frakturen identifizierten sie den Anprall des rotierenden Talus auf die tibiale Gelenkfläche, bedingt durch die auf den belasteten Fuß (Neutralstellung oder Plantarflexion) wirkenden Rotationskräfte. Bei weiterer Rotation des Talus kam es nach ihrer Definition zu einem zweiten posteromedialen Fragment, in der Regel in einem 45°-Winkel, welches sie als Mason Typ 2B klassifizierten. Mason-Typ-3-Frakturen sahen sie verursacht durch eine rein axiale Lastübertragung des Talus bei plantar-flektiertem Fuß. Begleitverletzungen der tibialen Gelenkfläche, des Malleolus medialis (knöchern und ligamentär), der Fibula sowie der Syndesmose wurden ebenfalls erfasst und ausgewertet. Die Interobserver-Reliabilität (2 Observer) zeigte mit einem Cohens-κ-Koeffizienten von 0,919 eine hohe Übereinstimmung [27].

Bei Mason-Typ-1-Frakturen wurde in 71 % der Fälle eine distale Fibulafraktur erfasst, in 78 % eine knöcherne oder ligamentäre Verletzung im Bereich des Malleolus medialis sowie in 100 % eine Verletzung der Syndesmose. Mason-Typ-2-Frakturen zeigten in 56 % der Fälle eine distale Fibulafraktur, in 96 % eine mediale knöcherne oder ligamentäre Verletzung sowie in 49 % eine Verletzung der Syndesmose. Mason-Typ-3-Frakturen wiesen dagegen meist hohe oder spiralförmige langstreckige Fibulafrakturen auf, wobei in 28 % der Fälle überhaupt keine Fibulafraktur diagnostiziert werden konnte. Dazu zeigte sich in 92 % der Mason-Typ-3-Frakturen eine mediale Verletzung, wobei diese in nur 4 % der Fälle rein ligamentär war. Eine Syndesmosenverletzung konnte bei den Mason-Typ-3-Frakturen nur in 20 % der Fälle diagnostiziert werden.

Bedingt durch die Korrelation mit dem Unfallmechanismus und den Begleitverletzungen, werteten Mason et al. ihre Klassifikation für so aussagekräftig, dass sie anhand der Klassifizierung Empfehlungen zur potenziellen operativen Versorgung stellten. Mason-Typ-1-Frakturen, extraartikulär und häufig zu klein für eine Schraubenosteosynthese, sollten intraoperativ aufgrund der in 100 % der Fälle vorliegenden Verletzung der Syndesmose diesbezüglich genauestens getestet werden, bei großzügiger Indikationsstellung zur Stabilisierung der Syndesmose. Mason-Typ-2A-Frakturen, dreieckförmig in die Inzisur einstrahlend und nur in etwa der Hälfte der Fälle mit einer Syndesmosenverletzung assoziiert, sollten direkt von dorsal über einen lateralen oder posterolateralen Zugang angegangen werden. Hiermit sollten eine bessere Kontrolle des Fragments und eine anatomische Reposition ermöglicht werden, um eine Stufe in der Inzisur und somit eine dorsale Fehlstellung der Fibula zu vermeiden. Zudem postulierten sie, dass durch die direkte Fixierung des posterioren Fragments eine weitere Stabilisierung der Syndesmose, im Falle einer Verletzung ebendieser, nicht mehr erforderlich sei. Im Fall von Mason-Typ-2B-Frakturen empfahlen Mason et al., das posteromediale Fragment aufgrund des Frakturverlaufes primär zu fixieren, da das posterolaterale Fragment ansonsten die Reposition des posteromedialen Fragments verhindern würde. Hierzu empfahlen die Autoren einen separaten posteromedialen Zugang. Bei Mason-Typ-3-Frakturen sahen Mason et al. aufgrund der Seltenheit von Syndesmosenverletzungen (20 %) die Notwendigkeit zur Stabilisierung wesentlich seltener. Als entscheidend sahen sie hier die hohe Rate an posteromedialen Verletzungen, weshalb sie diesbezüglich einen posteromedialen Zugang empfahlen.

Die Klassifikation nach Mason wird eher selten und ausschließlich in 5 englischsprachigen klinischen Studien verwendet (Tab. 7).

**Table 7:** 

Studie	Patienten	Inter‑/Inter-OR	Prädiktive Bedeutung
Gandham et al., 2020 [13]	*141 Patienten*	k. A.	k. A.
	Typ 1: 45 (32 %)		
	Typ 2A: 41 (29 %)		
	Typ 2B: 35 (25 %)		
	Typ 3: 20 (14 %)		
Mason et al., 2017 [27]	*121 Patienten*	Inter-OR von 0,919 Cohens-κ-Wert	k. A.
	Typ 1: 41 (34 %)		
	Typ 2A: 30 (25 %)		
	Typ 2B: 25 (20 %		
	Typ 3: 25 (21 %)		
Mason et al., 2019 [26]	*50 Patienten*	k. A.	Typ-3-Frakturen mit tendenziell schlechterem postoperativem Outcome (OMAS)
	Typ 1: 17 (34 %)		
	Typ 2A: 12 (24 %)		
	Typ 2B: 10 (20 %		
	Typ 3: 11 (22 %)		
Vosoughi et al., 2019 [46]	*47 Patienten*	k. A.	k. A.
	Typ 1: 0 (0 %)		
	Typ 2A: 0 (0 %)		
	Typ 2B: 47 (100 %)		
	Typ 3: 0 (0 %)		
Sun et al., 2021 [43]	*32 Patienten*	k. A.	Größe des „Die-punch“-Fragments ohne signifikanten Einfluss auf die postoperativen Ergebnisse
	Typ 1: 0 (0 %)		
	Typ 2A: 10 (31 %)		
	Typ 2B: 0 (0 %)		
	Typ 3: 22 (69 %)		

*k.* *A.* keine Angaben, *OMAS* Olerud-Molander Ankle Score,* OR* Observer-Reliabilität

## Diskussion

### Abgrenzung von Frakturen des posterioren Malleolus zu (posterioren) Pilon-Frakturen

Im klinischen Alltag bereitet die Abgrenzung von Pilon-Frakturen zu trimalleolären Sprunggelenkfrakturen oft Schwierigkeiten [17, 20, 35, 38]. Ein häufig angewandtes Differenzierungskriterium ist die Impression der tibialen Gelenkfläche im Rahmen einer Pilon-Fraktur. Unter anderem Bartonicek/Rammelt et al. konnten jedoch in ihrer Studie zeigen, dass alle Frakturen des posterioren Malleolus, inklusive der Frakturen außerhalb der Inzisur, Teile der Gelenkfläche der Tibia betrafen und häufig mit einer Impression der Gelenkfläche im Bereich der Hauptfrakturlinie einhergingen [4]. Unter den im Rahmen dieses Artikels betrachteten Klassifikationen definieren lediglich Bartonicek/Rammelt et al. ein klares Differenzierungskriterium der posterioren Malleolus-Fraktur zur Pilon-Fraktur. So wurde ein den gesamten Innenknöchel einschließender Frakturverlauf als Pilon-Fraktur definiert und aus der Studie ausgeschlossen [4].

Auch aufgrund dieser problematischen Differenzierung entwickelte sich in den letzten Jahren zusätzlich der Begriff der posterioren Pilon-Fraktur. 2013 veröffentlichten Klammer et al. die Ergebnisse einer retrospektiven Fallstudie von 11 Patienten und entwickelten anhand ihrer Daten eine Klassifikation für posteriore Pilon-Frakturen [21]. Im Jahre 2019 stellten Zhang et al. wiederum anhand der Auswertung von 36 weiteren Fällen eine eigene Klassifikation für posteriore Pilon-Frakturen auf [53]. Kritisch betrachtet, konnte die Einführung des Begriffs der posterioren Pilon-Fraktur und deren Klassifikation jedoch nicht die Problematik der Differenzierung von Pilon-Frakturen und trimalleolären Sprunggelenkfrakturen lösen, sondern verkomplizierte sie noch [8].

### Etablierung der Klassifikationen – Haraguchi, Bartonicek/Rammelt, Mason

Betrachtet man die Literatur zu Frakturen des posterioren Malleolus, wird mehrheitlich die Klassifikation nach Haraguchi et al. angewandt (Tab. 2). Dies beruht sicherlich zum einen auf der Tatsache, dass sie die älteste CT-basierte Klassifikation darstellt, zum anderen ist ihre Einteilung von den 3 dargestellten die übersichtlichste und problemlos auf alle Frakturen anzuwenden. Seit 2015 kristallisiert sich jedoch langsam eine Favorisierung der Klassifikation nach Bartonicek/Rammelt heraus (Tab. 5), wobei der offenkundigste Vorteil dieser Klassifikation in den sich ableitenden Therapieempfehlungen liegt. Die neueste Klassifikation nach Mason (Tab. 7) konnte bisher keine wirkliche Bedeutung erlangen. Alternative Klassifikationssysteme, wie die AO-Klassifikation, konnten sich im Bereich der posterioren Malleolus-Frakturen nicht etablieren. Weitere Studien zur Überprüfung der klinischen Anwendbarkeit der verschiedenen Klassifikation (Inter‑/Intraobserver-Reliabilität) sowie deren Aussagekraft über die Therapieempfehlung oder Prognose der verschiedenen Frakturtypen werden dringend benötigt.

### Vor- und Nachteile der verschiedenen Klassifikationen der posterioren Malleolus-Fraktur

**Figure 5:**
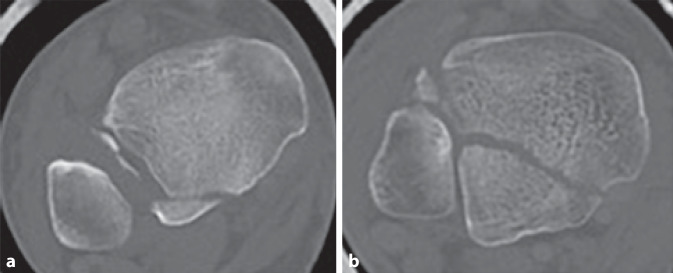

Haraguchi et al. konnten eine übersichtliche Klassifikation entwickeln, die es ermöglicht, alle Frakturen des posterioren Malleolus anhand ihrer Kriterien einzuteilen [14]. Jedoch ist ihr System nicht auf- oder absteigend bezüglich des Schweregrades oder der Komplexität der Fraktur aufgebaut, was die Kommunikation zwischen verschiedenen Ärzten ebenso wie die Planung der Therapie erschwert. Auch erfolgt ihre Einteilung nur anhand der axialen CT-Ebene und erfasst so nicht die vertikale Größenausdehnung der Fragmente. Ebenfalls nicht erfasst werden mediale Verletzungen und das Ausmaß der Beteiligung der Inzisur der Tibia (Notch). So umfasst der Typ I nach Haraguchi eine sehr große Bandbreite an Frakturen, von kleinen bis großen Fragmenten (Abb. 5). Die Differenzierung zu (posterioren) Pilon-Frakturen wird nicht definiert und somit auch nicht ausgeschlossen. Eine Therapieempfehlung lässt sich anhand der Klassifikation von Haraguchi et al. nicht ableiten.

**Figure 6:**
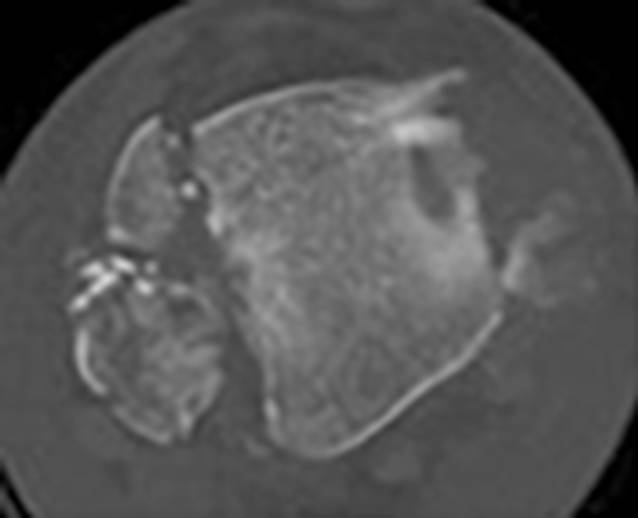

Bartonicek/Rammelt entwickelten ebenfalls eine übersichtliche Klassifikation, welche eine Einteilung aller Frakturen anhand ihrer Kriterien erlaubt [4]. Letztlich unklar bleibt jedoch Typ 5, welcher alle irregulären Frakturen subsumieren soll, die nicht den Typen 1–4 zuzuordnen sind (Abb. 6). Als einziges Klassifikationssystem definieren sie klar den Unterschied zu Pilon-Frakturen und schlossen diese aus ihrer Studie aus. Allerdings vermisst man eine klare Definition der Inzisur der Tibia (Notch) und verzweifelt mitunter an der Ein-Drittel-Grenze zwischen Typ-2- und Typ-4-Frakturen. Der Schweregrad der Frakturen ist aufsteigend, und es werden klare Therapieempfehlungen gegeben. Dies erklärt die zunehmende Verbreitung dieser Klassifikation für posteriore Malleolus-Frakturen.

Die Klassifikation nach Mason et al. ist eher unbekannt und findet wenig Anwendung [27]. In der Erläuterung ihres Klassifikationssystems greifen die Autoren auf schematische Zeichnungen zurück, die teilweise nicht mit der Beschreibung des Frakturtyps übereinstimmen. Auch wird die Inzisur der Tibia (Notch) nicht definiert. Die Einteilung ist so teilweise erschwert, gelingt jedoch letztlich für alle Frakturen. Auch Mason et al. leiten Therapieempfehlungen aus ihrem Klassifikationssystem ab, bleiben hier aber vage und letzten Endes sind die Empfehlungen nicht einheitlich umzusetzen.

**Figure 7:**
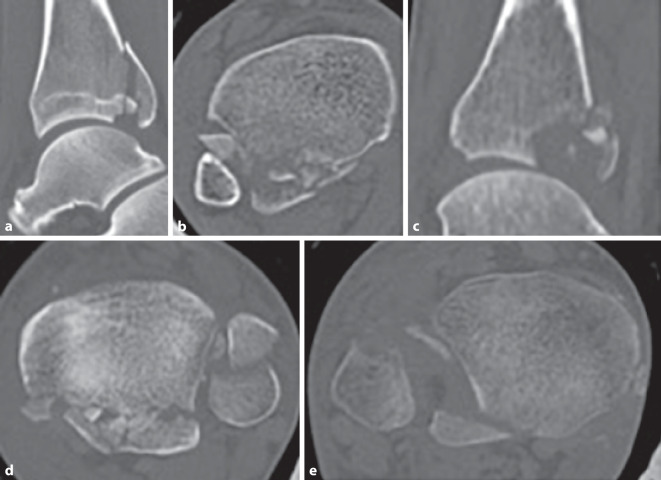

Letztlich beschreibt keine der Klassifikationen die Komplexität der posterioren Malleolus-Fraktur. Weder das Ausmaß der Gelenkflächenimpression, die Anzahl der Fragmente noch der Grad der Dislokation werden erfasst (Abb. 7). Fraglich ist jedoch, ob eine Erweiterung der bestehenden Klassifikationssysteme oder die Einführung eines neuen Systems, welches diese Faktoren zusätzlich berücksichtigen würde, zu komplex wäre, um eine suffiziente Kommunikation zwischen Ärzten (in verschiedenen Ausbildungsstufen) zu gewährleisten.

## Schlussfolgerung

Die Reposition und Osteosynthese dislozierter Frakturen des posterioren Malleolus stellen nicht nur die Gelenkfläche der Tibia sowie die Stabilität des tibiotalaren Gelenkes wieder her, sondern auch die Integrität der Inzisur (und ermöglichen damit die korrekte Einstellung der Fibula) und die Stabilität der tibiofibularen Syndesmose [4, 49]. Die ungünstigere Prognose von Trimalleolarfrakturen, verglichen mit anderen Frakturen im Bereich des oberen Sprunggelenks, ist allseits bekannt [11, 41, 45]. Prognostisch entscheidend sind hier v. a. die Qualität der Reposition bzw. das Geschick und die Erfahrung des Operateurs [34, 45].

Trotzdem besteht anhaltend kein Konsens bezüglich der Therapie der posterioren Malleolus-Frakturen. Dies beruht u. a. auf der Tatsache, dass sich bis heute keine Klassifikation durchsetzen konnte. Unter den vorgestellten Klassifikationen hat die Klassifikation nach Bartonicek/Rammelt das größte Potenzial, sich in Zukunft mehrheitlich durchzusetzen und so einen wesentlichen Anteil an der einheitlichen Therapie der herausfordernden Fraktur des posterioren Malleolus zu haben.

## Fazit für die Praxis

- Es besteht anhaltend kein Konsens bezüglich der Klassifikation und Therapie posteriorer Malleolus-Frakturen.
- Verschiedene CT-basierte Klassifikationssysteme wurden entwickelt, konnten sich jedoch noch nicht durchsetzen.
- Eine einheitliche, zuverlässige und möglichst umfassende Klassifikation würde zu einem besseren Verständnis der Fraktur führen, was letztendlich zu besseren postoperativen Ergebnissen führen könnte.
- Eine möglichst optimale Klassifikation würde auch die Kommunikation zwischen den verschiedenen Disziplinen (z. B. Unfallchirurgie und Radiologie) erleichtern.
- Aufgrund der klaren Definition und des aus der Klassifikation ableitbaren Therapiealgorithmus ist die Klassifikation nach Bartonicek/Rammelt am ehesten geeignet, sich in Zukunft durchzusetzen und weiter zu verbreiten.

## Supplementary Information





