# Supplementary material for: Established classification systems of posterior malleolar fractures: A systematic literature review
Source: Unfallchirurgie (Heidelb). 2022 Apr 8;126(5):387–98. [Article in German] doi: 10.1007/s00113-022-01162-3 (PMC10159979; doi:10.1007/s00113-022-01162-3)
Supplement: Supplementary file 1 [file 113_2022_1162_MOESM1_ESM.pdf]

Tab. 1 Modifizierter Coleman-Score.

Section	Number or factor	Score points
**Part A†**		
1. Study size: number of subjects (n)	>60	10
	41-60	7
	20-40	4
	<20	0
2. Mean follow-up (months)	>24	10
	12-24	7
	<12, not stated, or unclear	0
3. Number of different surgical procedures included in each reported outcome	One surgical procedure only	10
	More than one, but >90% of subjects undergoing the one procedure	7
	Not stated, unclear, or <90% of subjects undergoing the one procedure	0
4. Type of study	Randomized controlled trial	15
	Prospective cohort study	10
	Retrospective cohort study	0
5. Diagnostic certainty	In all	5
	In >80%	3
	In <80%, not stated, or unclear	0
6. Description of surgical procedure	Adequate (technique stated, and necessary details given)	10
	Fair (technique only stated without elaboration)	5
	Inadequate, not stated, or unclear	0
7. Description of postoperative rehabilitation	Well described with >80% of patients complying	5

	Well described with 60-80% of patients complying	3
	Not well described	0
**Part B‡**		
1. Outcome criteria	Outcomes measures clearly defined	2
	Timing of outcome assessment clearly stated	2
	Use of outcome tools that has reported good reliability	3
	Use of outcome tools with good sensitivity	3
2. Procedure for assessing outcomes	Subjects recruited (results not taken from surgeon's files)	5
	Investigator independent of surgeon	4
	Written assessment	3
	Completion of assessment with minimal investigator support	3
3. Description of subject selection process	Selection criteria reported and unbiased	5
	Recruitment rate reported >80%/<80%	5/3
	Eligible subjects not included in the study accounted for recruitment	5

†nur eine Anzahl von Punkten kann vergeben werden

‡mehrere Punkte können vergeben werden - wenn zutreffend
