# Supplementary material for: Established classification systems of posterior malleolar fractures: A systematic literature review
Source: Unfallchirurgie (Heidelb). 2022 Apr 8;126(5):387–98. [Article in German] doi: 10.1007/s00113-022-01162-3 (PMC10159979; doi:10.1007/s00113-022-01162-3)
Supplement: Supplementary file 2 [file 113_2022_1162_MOESM2_ESM.pdf]

Tab. 2. Beschreibung der 27 eingeschlossenen Studien inklusive der Ergebnisse des Coleman-Score.

Studie	Studientyp			Coleman-Fragebogen		
	prospektiv vs. retrospektiv	unizentrisch vs. multizentrisch	Evidenzlevel	Teil A	Teil B	Total
Bali et al, 2017 [1]	PS	MZ	IV	45	29	74
Bartonicek et al, 2015 [4]	RS	MZ	IV	38	23	61
Bartonicek et al, 2019 [3]	RS	MZ	IV	35	23	58
Blom et al, 2019 [7]	PS	UZ	I	58	35	93
Blom et al, 2020 [6]	PS	UZ	IV	40	35	75
Gandham et al, 2020 [13]	PS	UZ	IV	35	33	68
Haraguchi et al, 2006 [14]	PS	UZ	IV	65	33	98
He et al, 2020 [15]	RS	UZ	IV	50	28	78
Hendrickx et al, 2019 [16]	RS	UZ	IV	58	28	86
Huang et al, 2018 [18]	RS	UZ	III	53	28	81
Kostlivy et al, 2020 [22]	RS	UZ	IV	45	20	65
Maluta et al, 2021 [24]	RS	UZ	IV	66	28	94
Mangnus et al, 2015 [25]	PS	UZ	IV	59	22	81
Mason et al, 2017 [27]	RS	UZ	III	73	33	106
Mason et al, 2019 [26]	RS	UZ	IV	74	35	109
Meijer et al, 2015 [31]	RS	UZ	IV	59	27	86

Meijer et al, 2016 [30]	RS	UZ	IV	54	25	479
Mertens et al, 2020 [32]	PS	UZ	IV	74	35	109
Mitchell et al, 2019 [33]	RS	UZ	IV	47	22	69
Neumann & Rammelt, 2021 [37]	RS	UZ	IV	75	30	70
Quan et al, 2021 [40]	RS	UZ	IV	48	23	71
Sultan et al, 2020 [42]	RS	UZ	IV	53	23	76
Sun et al, 2021 [43]	RS	UZ	IV	59	20	79
Vosoughi et al, 2019 [46]	PS	UZ	IV	54	22	76
Yang et al, 2020 [50]	RS	UZ	IV	59	20	79
Yi et al, 2018 [51]	RS	UZ	IV	47	22	69
Yu et al, 2021 [52]	PS	UZ	IV	90	35	125

MZ multizentrisch, ***RS*** retrospektiv, ***UZ*** unizentrisch
